# Semaphorins and Their Receptors in Hematological Malignancies

**DOI:** 10.3389/fonc.2019.00382

**Published:** 2019-05-09

**Authors:** Li Wei, Hongbo Li, Luca Tamagnone, Hua You

**Affiliations:** ^1^Affiliated Cancer Hospital & Institute of Guangzhou Medical University, Guangzhou, China; ^2^Department of Pulmonary and Critical Care Medicine, Taihe Hospital, Hubei University of Medicine, Shiyan, China; ^3^Istituto di Istologia ed Embriologia, Università Cattolica del Sacro Cuore, Rome, Italy; ^4^Fondazione Policlinico Universitario Agostino Gemelli, IRCCS, Rome, Italy; ^5^YouJiang Medical University For Nationalities, Baise, China; ^6^Affiliated Hospital of Academy of Military Medical Sciences, Beijing, China

**Keywords:** semaphorins, Neuropilin, Plexin, leukemia, lymphoma, multiple myeloma

## Abstract

While semaphorins were initially identified as axonal guidance cues for wiring the neural network, it was then recognized their wide relevance in tissue development and homeostasis. Notably, semaphorin activities were also extensively studied in many types of solid tumors; however, their relevance in hematological malignancies is far from understood. In this mini-review, we surveyed the current knowledge about semaphorins and their receptors in leukemias, lymphomas, and multiple myeloma. Noteworthy, current data support a promoting role for Semaphorin 4D and Neuropilin-1 in these tumors, while Semaphorin 3A seems to consistently act as oncosuppressor in leukemias and multiple myeloma. The expression levels and functional activities of SEMA3B, SEMA3F, and Neuropilin-2 have furthermore been investigated in leukemias and lymphoma cells. Herein, we reviewed the state of the art and highlighted some of the open questions to be addressed in the field.

## Introduction

The first Semaphorin was identified as repelling molecule for axonal guidance in the developing nervous system (1). Thereafter, the functional diversity of semaphorin family members beyond the wiring of neuronal network has been widely explored, ranging from the regulation of cardiovascular development and angiogenesis, to bone homeostasis and immune response (2, 3). Furthermore, while semaphorin activities have been extensively studied in many types of solid tumors (4, 5), their relevance in hematological malignancies is far from understood. The semaphorin family includes both secreted and transmembrane proteins, which can signal in autocrine/paracrine manner or upon cell-cell contact, respectively. Notably, semaphorin receptors (especially found in Neuropilin and Plexin families) can form a range of diverse complexes with distinctive signaling cascades, and interact with other growth factor receptors, making semaphorin activities highly cell-context dependent (6). Moreover, transmembrane semaphorins can mediate bidirectional signals, acting both as ligands and as receptors (7). In this mini-review, we summarized the current knowledge about semaphorins and their receptors in hematological malignancies, including leukemia, lymphoma and multiple myeloma, and highlighted the main research trends and open questions to be addressed in the field.

## Semaphorins in Leukemias

Leukemias are a group of life threatening malignant disorders of the blood and bone marrow (BM), presenting with increased numbers of leucocytes and BM precursors. The dominantly presenting leukemia cells may be mature such as in chronic lymphocytic leukemia (CLL), or precursor cells of various lineage such as in the acute leukemias, or both precursor and mature cells as in chronic myeloid leukemia (CML) (8). The first report about semaphorins and their receptors in leukemias concerns transmembrane Semaphorin 4D (SEMA4D/CD100) and dates back in 2003 (9) (Table 1). SEMA4D/CD100 was found to be expressed in B-cell chronic lymphocytic leukemia (CLL) cells and in normal CD5^+^ B lymphocytes, and its high-affinity receptor Plexin-B1 was found in BM stromal cells, follicular dendritic cells, and activated T lymphocytes. This expression pattern led to explore a potential interaction in trans between CD100^+^ lymphocytes/CLL cells and neighboring Plexin-B1^+^ cells in the microenvironment. It was discovered that this interaction enhanced the proliferation and extended the life span of both leukemic and normal CD5^+^ B cells (9). Consistent with these findings, a subsequent independent study demonstrated that an upstream CD31-CD38 signaling axis in trans between stromal and leukemic cells of CLL patients actually led to SEMA4D/CD100 up-regulation, promoting leukemia cell viability through Plexin-B1 found in stromal cells, as well as a concomitant decrease in the expression of CD72 negative regulator of immunity (10). In both studies, however, it remained undetermined the identity of pro-survival factors produced by stromal cells in response to SEMA4D/Plexin-B1 signaling. Another study assessed the growth-suppressing activity of CD72, the low affinity receptor of SEMA4D, in acute myelogenous leukemia (AML) cells (11). In fact, CD72 ligation by an agonistic antibody, or by its natural ligand SEMA4D/CD100, suppressed the proliferation of the AML Kasumi-1 cells and induced apoptotic cell death. The implicated molecular mechanism depends on CD72 phosphorylation and SHP-1 recruitment, leading to de-phosphorylation of Src family kinases and JNK (11). Thus, multiple data support a pro-tumorigenic role of SEMA4D in leukemias, consistent with the current knowledge about this semaphorin in solid tumors (12).

**Table 1:** Summary of the expression profile of semaphorins and receptors in hematological malignancies.

**Semaphorins, receptors**	**Target cells**
SEMA3A	**↓**acute lymphoid and myeloid leukemia (ALL/AML) cells (18) **↓**chronic myelogenous leukemia (CML) cells (18) **↓**the serum of ALL and AML patients (19) **↓**bone marrow endothelial cells isolated from patients with multiple myeloma (40) **↓**the serum level of multiple myeloma patients (19)
SEMA3B	**↑**the circulating blood of AML patients (33)
Neuropilin-1 (NRP1)	**↑**bone marrow biopsies of AML patients (25) **↑**CLL cells (26)
SEMA3F and its receptor, Neuropilin-2 (NRP2)	**↑**human T-cell acute lymphoblastic leukemia/lymphoma primary cells (35)
SEMA4D/CD100	**↑** B-cell chronic lymphocytic leukemia (CLL) cells (9) **↑** normal CD5^+^ B lymphocytes (9) **↑**T-cell non-Hodgkin's lymphoma (NHL) (37) ↓B-cell lymphomas (small lymphocytic lymphoma-SLL, follicular lymphomas, marginal zone lymphoma, mantle cell lymphoma, and diffuse large B-cell lymphoma) (37) **↑**bone marrow and the serum of multiple myeloma patients (40)
High-affinity Sema4D-Receptor, PLEXIN-B1	**↑**bone marrow stromal cells (9) **↑**follicular dendritic cells (9) **↑**activated T lymphocytes (9) **↑**bone marrow and the serum of multiple myeloma patients (40)
Low affinity Sema4D-Receptor, CD72	**↑**acute myelogenous leukemias (AMLs) cell line (Kasumi-1) (11)

*↑, increased levels or prominent basal expression; ↓, decreased expression (compared to normal)*.

Notably, SEMA4D-targeting agents for prospective application in clinical trials have been developed (13). VX15/2503 is a humanized IgG4 monoclonal antibody that binds specifically to SEMA4D and blocks the binding of SEMA4D to its receptors, plexinB1, plexinB2, and CD72 (14, 15). A total of 42 patients with advanced refractory solid tumors were enrolled, weekly i.v. doses were administered on a 28-day cycle, and safety, immunogenicity, pharmacokinetics (PK), efficacy, cSEMA4D expression and saturation, soluble SEMA4D (sSEMA4D) serum levels, and serum biomarker levels were evaluated (15). Patients experienced well tolerances for VX15/2503, one patient (20 mg/kg) experienced a partial response, 45.2% (19/42) of patients exhibited stable disease for 8 weeks, and 19% (8/42) of patients exhibited stable disease for 16 weeks (15). Recent studies in mouse models also demonstrated that targeting SEMA4D improved response to immune checkpoint blockade via attenuation of myeloid-derived suppressor cells (MDSC) recruited in the tumor microenvironment (16, 17).

The expression of the secreted semaphorin SEMA3A was reported to be lower in acute lymphoid and myeloid leukemia (ALL/AML) and chronic myelogenous leukemia (CML) cells, compared to hematopoietic cells found in the normal BM (18). In a recent study, it was also reported that SEMA3A levels in the serum of ALL and AML patients are significantly reduced compared to healthy individuals (19). Consistent with a putative suppressor role of this semaphorin, it was interestingly shown that SEMA3A (via receptor complexes including Neuropilin-1/NRP1 and Plexin-A1) can promote Fas translocation into membrane rafts, thus sensitizing leukemic cells to Fas-mediated apoptosis (20). In an independent study, SEMA3A was also found to induce apoptosis in leukemia cells, via NRP1 co-receptor (14). It was furthermore shown that SEMA3A may partially reverse VEGF-induced proliferation of acute myeloid leukemia (AML) cells, and the combined administration of VEGF inhibitors and recombinant SEMA3A was suggested as potential treatment for AML patients (21). Notably, the potential application of recombinant SEMA3A (or derived molecules) in cancer therapy has been studied in multiple pre-clinical models of solid tumors, mainly focusing on its ability to inhibit endothelial cells sustaining aberrant angiogenesis (22–24).

Interestingly, the levels of the semaphorin receptor Neuropilin-1 (NRP-1) in BM biopsies of patients with newly diagnosed, untreated, AML were significantly increased compared those found in normal controls; and in fact higher level of NRP-1 were associated with poor overall survival (25). NRP1 was found to be highly expressed also in CLL cells, and its expression was upregulated by VEGF (26). Interestingly, it has been shown that NRP1 transcripts are targeted by miR-9 that was found to be downregulated in ALL cells; indeed miR-9 forced re-expression could inhibit leukemia cell proliferation and cell cycle progression (27). In addition, clinical studies revealed high NRP1 levels to be an independent risk factor for poor overall survival of AML patients, as well as a predictor of shorter recurrence-free survival after complete therapeutic response in AML (28). Moreover, it was demonstrated that a cell-internalized oligopeptidic molecule targeting NRP1 displays potent anti-leukemia cell effect (29). In general, NRP1 expression has been associated with cancer progression in multiple types of solid tumors (30), and diverse NRP1-targeting agents have been demonstrated to have tumor suppressing activity in mouse preclinical models (31, 32).

Different from SEMA3A, the levels of SEMA3B in the circulating blood of AML patients were significantly increased compared with those of healthy controls (33). Actually, the role of SEMA3B in cancer is controversial, being considered as a suppressor or as a promoter in different studies (34). Moreover, SEMA3F and its receptor Neuropilin-2/NRP2 were found to be highly expressed in human T-cell acute lymphoblastic leukemia/lymphoma primary cells, but SEMA3F was shown to inhibit the migration of malignant cells induced by CXCL12 and S1P (35).

## Semaphorins in Lymphomas

Lymphoma is a heterogeneous group of hematological malignancies derived from lymphocytes, with various underlying etiological factors, clinical manifestations, histopathological features, and genetic/molecular profiles; they may thus be subject to different therapeutic strategies. Lymphomas can be divided into two major categories: Hodgkin's lymphomas and non-Hodgkin's lymphomas (NHL). The latter constitute around 90% of the lymphoid neoplasms, and can be classified into B and T/natural killer (NK)-cell lymphomas (36). Dorfman and coworkers examined SEMA4D/CD100 expression pattern in 138 cases of non-Hodgkin's lymphoma (NHL) by immunohistochemistry (37); most of T-cell NHL cases were positive for SEMA4D, whereas B-cell lymphomas were consistently negative for SEMA4D, including cases of small lymphocytic lymphoma (SLL)/chronic lymphocytic leukemia (CLL), follicular lymphomas, marginal zone lymphoma, mantle cell lymphoma, and diffuse large B-cell lymphoma. These results were further confirmed by analyzing gene expression at mRNA level, however, the signaling mechanism and functional role of SEMA4D in T-cell lymphoma cells was not clarified in this study.

SEMA3F was shown to inhibit normal thymocytes as well as T-cell lymphoblastic lymphoma (T-LBL) primary cells migration induced by CXCL12 and S1P (35). Notably T-LBL samples were found to express high levels of both SEMA3F and its receptor Neuropilin-2 (NRP2), although the functional relevance of these molecules was not addressed by loss-of-function experiments, in this study. SEMA3F is generally considered as a tumor suppressor gene and its expression is often downregulated in solid tumors (34), but its function could be different in leukemia and lymphomas, and awaits clarification. NRP2 is considered a potential therapeutic target in certain solid tumors, and blocking antibodies and other targeting tools have been validated for this purpose in preclinical mouse models (38, 39). Notably, the homologous semaphorin co-receptor NRP1, was successfully targeted in lymphoma cells with an oligopeptidic drug candidate for cancer therapy (29).

## Semaphorins in Multiple Myeloma

Despite improvements in the therapeutic protocols, multiple myeloma (MM) remains an incurable disease, due to the proliferation of malignant plasma-cells in the bone marrow (BM), and accumulation of monoclonal antibodies in the blood and urine, with associated organ dysfunction (19, 39, 40). MM is thought to evolve from a monoclonal gammopathy of undetermined significance (MGUS) (19). SEMA3A was the first family member found to have a functional role in MM (40). In fact, BM endothelial cells isolated from patients with MM showed higher levels of VEGF and lower levels of SEMA3A compared to normal controls, consistent with the loss of endothelial inhibitory activity attributed to SEMA3A (40). These results underscored the potential usefulness of SEMA3A as anti-angiogenic molecule to restore the physiological regulation in the microenvironment. Consistently, a subsequent study demonstrated that the concentration of SEMA3A in serum of MM patients was strongly reduced, compared to healthy individuals, and the extent of this reduction was significantly associated with disease progression (19). In a preclinical experimental model in mice, it was demonstrated that forced SEMA3A overexpression in MM cells could inhibit disease progression, reduce the incidence and severity of bone lesions, and prolong overall survival.

Another study reported about increased levels of SEMA4D and its receptor Plexin-B1 in both BM and the serum of MM patients (41). Notably, elevated SEMA4D levels correlated with increased bone resorption, hypercalcemia, and higher stage, providing a potential target for novel therapeutic approaches in MM (41). It was then mechanistically investigated the relevance of SEMA4D in myeloma cells by knock down experiments (42); notably, SEMA4D expression was found to mediate paracrine signaling preventing the induction of the pivotal differentiation factor Runx2 in osteoprogenitor cells. Moreover, myeloma cell-conditioned medium induced SEMA4D expression in osteoclasts, featuring an additional paracrine mechanism to inhibit osteoblasts activity. Thus, SEMA4D signaling in myeloma cells seems to act both directly and indirectly to hinder bone formation (42). In sum, as discussed above with reference to leukemias, SEMA4D could be a promising therapeutic target also for the treatment of multiple myeloma.

## Conclusions and Future Perspectives

Accumulating evidences underscore the multifaceted activities of semaphorins and their receptors in hematological malignancies (Table 1). Different from solid tumors, the regulation of leukemia and lymphoma cells may occur in the circulation, as well as in BM and lymphoid organs, which are poorly understood tumor microenvironments. Thus, future studies are warranted to elucidate the molecular mechanisms of semaphorin activity in this context, and validate the potential clinical application of these molecules as prognostic/predicting factors, as well as novel targets for therapy. Notably, a SEMA4D-targeting monoclonal antibody, VX15/2503, is under validation in clinical trials for patients with advanced solid tumors refractory to therapy. Indeed, targeting SEMA4D with this antibody in murine models fostered a shift toward pro-inflammatory signals and anti-tumor immune response within the microenvironment; and the combination of anti-SEMA4D and anti-CTLA-4 antibodies achieved synergistic progress of tumor rejection and mice survival (16, 17). These data open perspectives for future application of anti-SEMA4D antibodies (or other semaphorin-targeting molecules) in the fight to cure hematological malignancies.

## Author Contributions

LW and HL searched for current literature on the topic and drafted the manuscript outline. LT and HY reviewed the manuscript and finalized it for publication.

### Conflict of Interest Statement

The authors declare that the research was conducted in the absence of any commercial or financial relationships that could be construed as a potential conflict of interest.
